# D-carvone attenuates LPS-induced acute lung injury via TLR4/NF-κB and Nrf2/HO-1 signaling pathways in rats

**DOI:** 10.1007/s00210-025-04024-y

**Published:** 2025-03-21

**Authors:** Nergis Ulaş, Hilal Üstündağ, Seçkin Özkanlar, Elif Erbaş, Adem Kara, Yunusemre Özkanlar

**Affiliations:** 1 Department of Internal Medicine, Faculty of Veterinary Medicine, Ataturk University, Erzurum, Turkey; 2 Department of Physiology, Faculty of Medicine, Erzincan Binali Yıldırım University, Erzincan, Turkey; 3 Department of Biochemistry, Faculty of Veterinary Medicine, Ataturk University, Erzurum, Turkey; 4 Department of Histology and Embryology, Faculty of Veterinary Medicine, Atatürk University, Erzurum, Turkey; 5 Department of Molecular Biology and Genetics, Faculty of Science, Erzurum Technical University, Erzurum, Turkey; 6 Department of Internal Medicine, Faculty of Veterinary, Ondokuz Mayis University, Samsun, Turkey

**Keywords:** Sepsis, Lung injury, D-carvone, Inflammation, Apoptosis, Oxidative stress, Cytokines

## Abstract

Acute lung injury (ALI) is a severe respiratory disorder associated with high morbidity and mortality. Lipopolysaccharide (LPS) is widely used to induce ALI in animal models. D-carvone, a natural monoterpene, has been reported to possess anti-inflammatory and antioxidant properties. This study aimed to investigate the protective effects of D-carvone on LPS-induced ALI in rats. Thirty-six male rats were randomly divided into six groups (*n* = 6): control, D-carvone (10 mg/kg and 20 mg/kg p.o.), LPS (10 mg/kg *E. coli* lipopolysaccharide i.p.), and LPS + D-carvone (LPS with either 10 or 20 mg/kg D-carvone). D-carvone was administered orally once daily for 10 days. On day 10, sepsis was induced with LPS administration, and samples were collected after 6 h under deep anesthesia. LPS administration caused significant lung injury, as evidenced by increased histopathological scores, upregulation of pro-inflammatory markers (TLR4, IL-1β, TNF-α), and oxidative stress (increased MDA, decreased GSH and SOD). Treatment with D-carvone at both doses significantly attenuated these changes. D-carvone downregulated pro-inflammatory markers, upregulated anti-inflammatory (NRF2) and anti-apoptotic (Bcl-2) proteins, and reduced the levels of pro-inflammatory cytokines (IL-1β, TNF-α, IL-8) in lung tissues. In conclusion, D-carvone protects against LPS-induced ALI in rats, possibly through its anti-inflammatory and antioxidant properties. These findings suggest that D-carvone could be a potential therapeutic candidate for preventing and treating ALI.

**Graphical Abstract:**

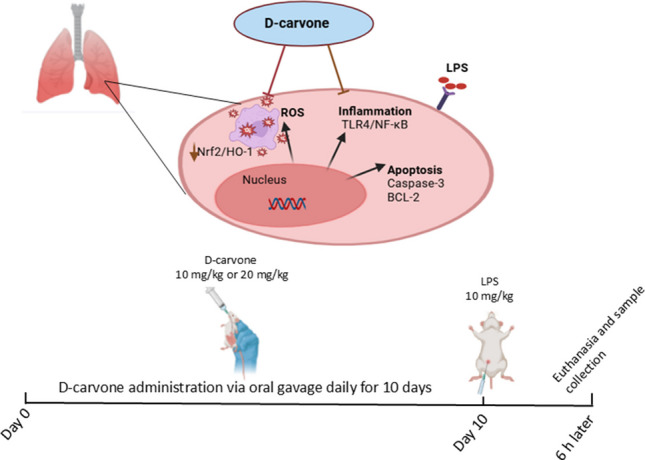

## Introduction

Acute lung injury (ALI) and its more severe form, acute respiratory distress syndrome (ARDS), are life-threatening conditions characterized by acute onset of hypoxemia, noncardiogenic pulmonary edema, and diffuse alveolar damage (Fan et al. 2018). Despite advances in supportive care and mechanical ventilation strategies, ALI/ARDS remains a significant cause of morbidity and mortality in critically ill patients, with an estimated mortality rate of 30–40% (Bellani et al. 2016). The pathogenesis of ALI/ARDS involves a complex interplay of inflammatory mediators, oxidative stress, and cellular damage, leading to increased vascular permeability, alveolar-capillary barrier disruption, and impaired gas exchange (Thompson et al. 2017).

Lipopolysaccharide (LPS), a key component of the outer membrane of gram-negative bacteria, is widely used to induce experimental ALI in animal models (Matute-Bello et al. 2008). LPS binds to Toll-like receptor 4 (TLR4) on immune cells, triggering a cascade of inflammatory responses, including the activation of nuclear factor-κB (NF-κB) and the release of pro-inflammatory cytokines such as interleukin-1β (IL-1β), tumor necrosis factor-α (TNF-α), interleukin-6 (IL-6), and interleukin-8 (IL-8) (Jiang et al. 2018; Zeng et al. 2024). These inflammatory mediators recruit neutrophils to the lungs, producing reactive oxygen species (ROS) and further exacerbating tissue damage (Matthay et al. 2019). Additionally, LPS-induced oxidative stress contributes to the pathogenesis of ALI by causing lipid peroxidation, protein oxidation, and DNA damage, ultimately leading to cell death and organ dysfunction (Kellner et al. 2017).

Current therapeutic strategies for ALI/ARDS primarily focus on supportive care, including mechanical ventilation, fluid management, and treatment of underlying infections (Keddissi et al. 2018; Vellingiri et al. 2020). However, there is a pressing need for effective pharmacological interventions that can modulate the inflammatory response and attenuate lung injury. In recent years, natural products have gained increasing attention as potential therapeutic agents for ALI/ARDS due to their anti-inflammatory and antioxidant properties (Aboushanab et al. 2021; Üstündağ, 2023; Üstündağ et al. 2023a).

Carvone (p-mentha-6,8-dien-2-one) is a natural unsaturated monoterpene found as a major constituent in the essential oil of some aromatic medicinal plants, such as caraway and spearmint (De Carvalho & Da Fonseca 2006). Two enantiomeric forms of carvone (*L*-carvone and *D*-carvone) have been identified that vary in their pharmaceutical values (Ye et al. 2022). This variation may be explained by the stereoselective metabolism of carvone enantiomers by the liver microsome (Jäger et al. 2000). D-carvone enantiomer has shown a wide range of pharmacological effects including immunomodulatory, anti-tumorigenic, chemo-preventive, anti-hyperlipidemic, and anti-hypertensive (Gopalakrishnan et al. 2019; Lv et al. 2021; Asle-Rousta et al. 2023). In a study, Moço et al. demonstrated that D-carvone and its chemical derivatives exhibited significant anti-inflammatory properties by reducing nitric oxide production and modulating pro-inflammatory mediators in LPS-stimulated macrophages (Moço et al. 2023). Another study by Chen et al. showed that D-carvone (30 and 60 mg/kg) exhibited significant anti-arthritic activity in complete Freund’s adjuvant-induced arthritis by modulating inflammatory cytokines and improving antioxidant status (Chen et al. 2020). Furthermore, Dai et al. reported that D-carvone (10 and 20 mg/kg) protected against cerebral ischemia–reperfusion injury by attenuating neuroinflammation through the TLR4/NLRP3 signaling pathway and reducing pro-inflammatory cytokines IL-1β and TNF-α (Dai et al. 2020). The protective effects of D-carvone have been previously reported in endotoxemic rats (Zhao and Du 2020). However, to our knowledge, no previous studies have investigated the underlying molecular mechanisms of D-carvone’s protective effects against LPS-induced lung injury.

The current study aimed to investigate the protective effects of D-carvone against LPS-induced acute lung injury in rats and to elucidate its underlying molecular mechanisms, particularly focusing on TLR4/NF-κB-mediated inflammatory pathways, Nrf2/HO-1-dependent antioxidant responses, and the modulation of apoptotic signaling through the Bcl-2/Caspase-3 axis.

## Materials and methods

### Animal experiments

Thirty-six male rats (2 months old, weighing 150–200 g) were used in this study. The animals were randomly divided into six groups (*n* = 6 per group): healthy (control), LPS, D-carvone 10 mg/kg (D-Car 10), D-carvone 20 mg/kg (D-Car 20), LPS + D-Car 10, and LPS + D-Car 20. All animals were maintained under standard laboratory conditions with a 12-h light/dark cycle and provided with a standard rodent diet and water ad libitum. The use of animals and all experimental protocols were approved by the Ataturk University Animal Experiments Local Ethics Committee (Decision No: 227, Meeting No: 2023/14, dated December 25, 2023).

### Chemicals and drugs

LPS (*Escherichia coli* O55:B5, Santa Cruz Biotechnology Inc, Lot No: H0522) was administered i.p at a 10 mg/kg dose to induce ALI (Gao et al. 2022). The solution preparation involved the dissolution of 10 mg of lyophilized LPS powder in 10 mL of 0.9% normal saline. This mixture was then subjected to a 30-min vortexing process to ensure complete dissolution before use. D ( +)-Carvone (Purity-96%, natural, liquid solution) was purchased from Thermoscientific (Cat No:150680250) and stored at a temperature of 2–4 °C, protected from sunlight. Following storage, D-carvone was administered orally via gavage at doses of 10 or 20 mg/kg (Dai et al. 2020).

### Experimental design

The healthy control group received no treatment. The LPS group received 10 mg/kg *E. coli* lipopolysaccharide (i.p.). The D-Car groups received either 10 mg/kg or 20 mg/kg D-carvone via oral gavage daily for 10 days. The LPS + D-Car groups received both LPS (10 mg/kg, i.p.) and D-carvone (10 or 20 mg/kg, p.o.) treatments. On day 10, sepsis was induced in the LPS groups, and samples were collected 6 h post-LPS administration (Guo et al. 2022).

### Sample collection and analysis

At the end of the experimental period, all animals were euthanized under deep anesthesia with a combination of ketamine (100 mg/kg) and xylazine (15 mg/kg) administered intraperitoneally (Üstündağ et al. 2023b). Following anesthesia, the animals were euthanized by cervical dislocation, and samples, including lung tissues and blood, were collected for analysis. Blood samples were collected in vacuum tubes, kept at room temperature for 20 min for coagulation, and then centrifuged at 1500 × g for 15 min. The sera samples were transferred to 1.5-mL Eppendorf tubes and stored at − 80 °C. The right lung tissue was promptly stored at − 80 °C for subsequent biochemical analyses. The left lung was fixed in 10% formalin for histopathological examination using standard tissue processing techniques.

### Histopathological analyses

Lung tissues were fixed in 10% neutral formalin solution for 72 h, with the solution being refreshed every 24 h. The tissues were then processed through graded alcohol and xylol series and embedded in paraffin blocks. Sections of 5 µm thickness were cut using a microtome (Leica RM2125 RTS) for histopathological evaluations. The sections were stained using Crossman’s Modified Mallory’s Triple Stain method and examined under a light microscope for tissue damage assessment. A computer and camera-equipped light microscope (Zeiss AXIO Scope.A1) was used for microscopic examination.

### Histopathological evaluation

**Fig. 1:**
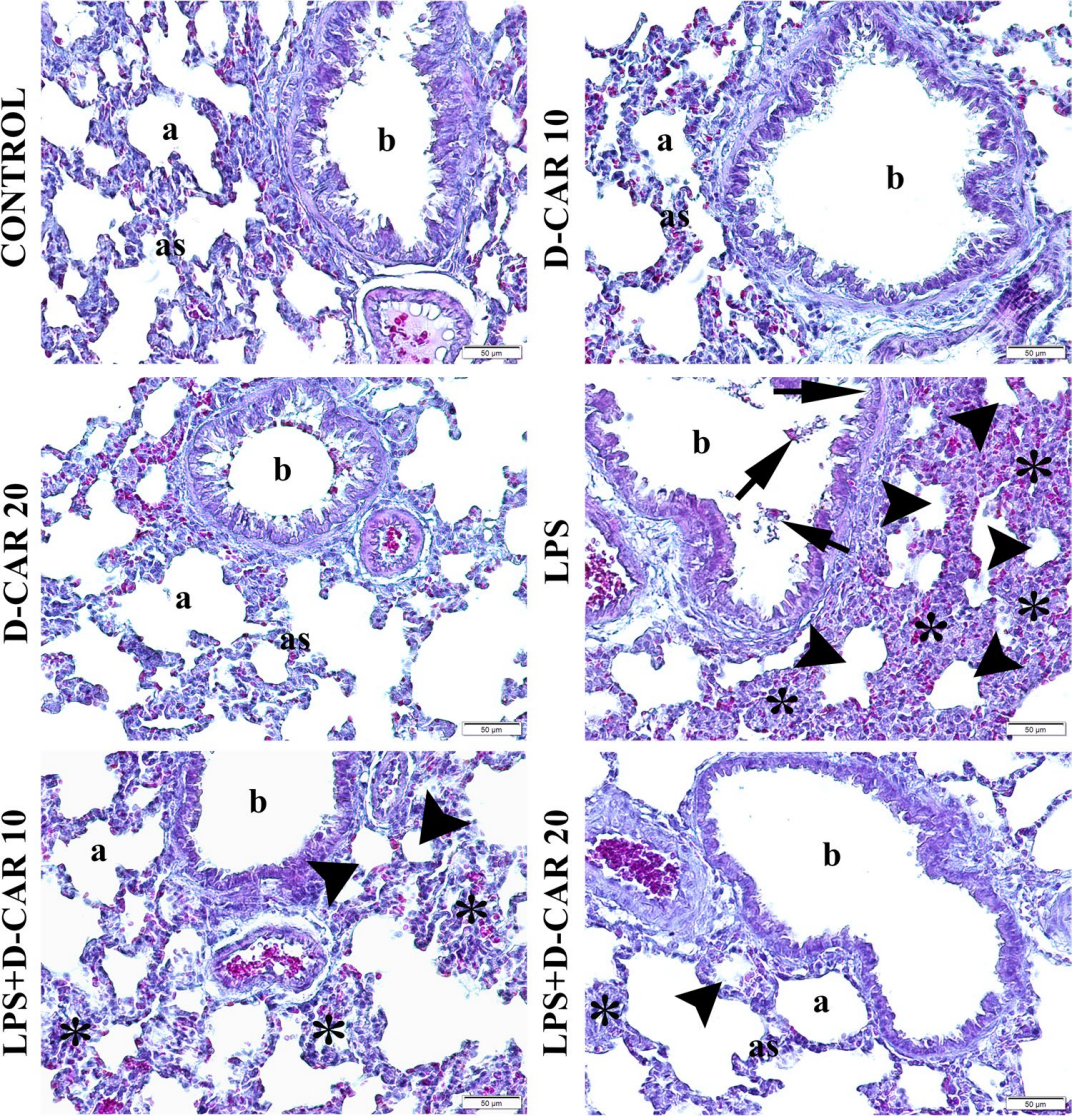
Micrographs of lung tissues of all groups (Mallory’s triple staining as modified by Crossman, magnification × 200). b, bronchiole; a, alveolus; as, alveolar septa; asterisk, thickening in alveolar septa; arrowhead, degenerative alveolus; arrow, degeneration in bronchiole epithelium

**Fig. 2:**
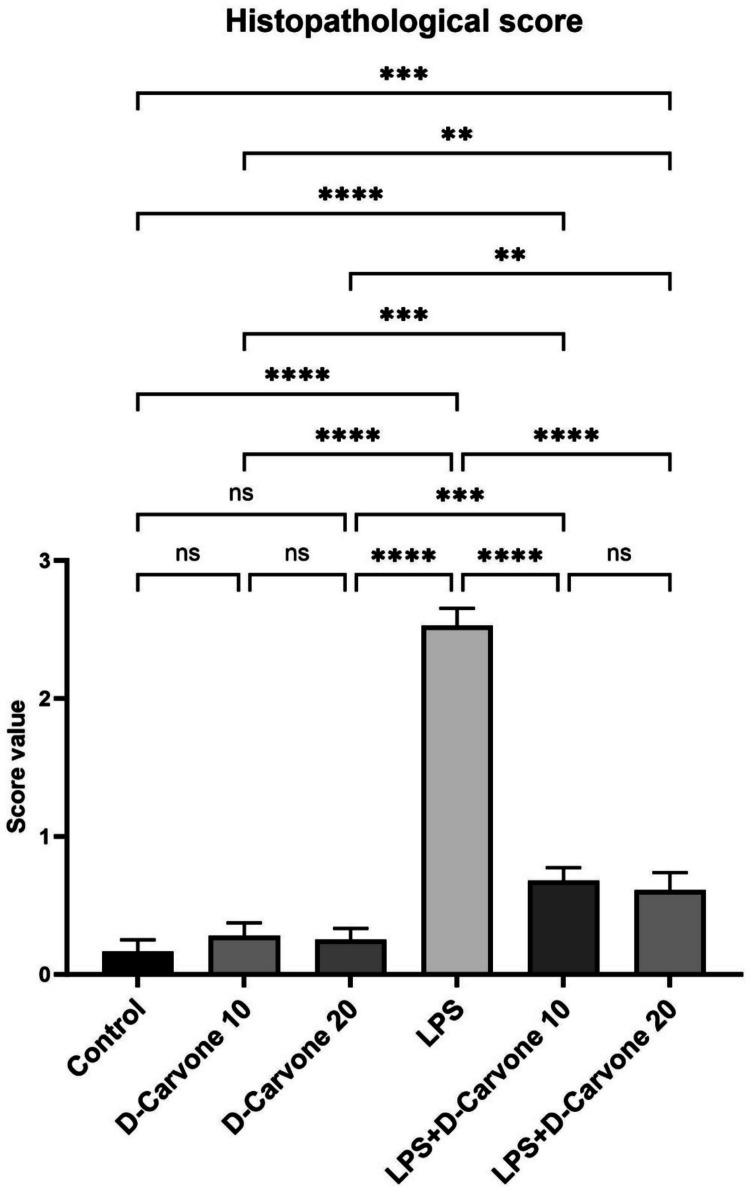
Evaluation of lung tissue histology. Values are given as mean ± SD (*n* = 6) and analyzed by one-way ANOVA followed by Tukey’s test. Statistical significance between groups is indicated as follows: ns (not significant, *p* > 0.05), * (*p* < 0.05), ** (*p* < 0.01), *** (*p* < 0.001), and **** (*p* < 0.0001)

Lung tissue sections were evaluated for pulmonary edema, vascular and alveolar structure, and bronchiolar pathology using a scoring system of 0 (normal), 1 (mild), 2 (moderate), and 3 (severe). The assessment criteria, adapted from Passmore et al. and Erbaş et al., are presented in Table 1 (Passmore et al. 2018). The obtained data are presented in Figs. 1 and 2.

**Table 1:** Lung histopathology scoring criteria

Score	Vascular features	Extravascular and alveolar formations	Bronchiolar features
0	Normal	Normal	None
1	Few erythrocytes in the interstitium	Mild inflammation	Minimal inflammation
2	Vascular obstruction, moderate hemorrhage	Moderate inflammation, moderate thickening of alveolar septa	Moderate inflammation, focal degeneration in bronchiolar walls
3	Severe hemorrhage and severe vascular obstruction	Severe inflammation, advanced thickening of alveoli	Advanced degeneration in bronchiolar epithelium

### Western blotting

Lung tissues were stored at − 80 °C until western blot analysis. After weighing, the tissues were pulverized under liquid nitrogen and subjected to lysis using radioimmunoprecipitation (RIPA) buffer (Ecotech Bio, Turkey) supplemented with protease and phosphatase inhibitors. The samples were then homogenized using a tissue disruptor (Qiagen, USA) for 20 s at 30 Hz. This process enabled the assessment of protein expressions for TLR4, HO-1, NRF2, IL-1β, TNF-α Bcl-2, and Caspase-3. The total protein concentration in the lung tissue was quantified using a protein assay kit (Pierce BCA, Thermo Scientific, USA). The antibodies utilized in this study are listed in Table 2. For protein separation, 30 µg of protein was subjected to 10% SDS-PAGE and subsequently transferred onto a PVDF membrane. The membranes were blocked with 5% bovine serum albumin for 90 min at room temperature and then incubated overnight at 4 °C with the appropriate primary antibodies, followed by washing with TBST and incubation with a horseradish peroxidase-conjugated secondary antibody (Santa Cruz, sc-2004/sc-2005) for another 90 min. Protein bands were detected and analyzed using Image Lab™ Software (Bio-Rad, Hercules, CA, USA) and the enhanced chemiluminescence reagent Western ECL substrate (Thermo, 3405).

**Table 2:** Antibodies used in Western analyses

Antibody	Catalog no	Western dilution
TLR4	AF7455, Affinity Biotech	1/1000
HO-1	AF5393, Affinity Biotech	1/1000
NRF2	BF8017, Affinity Biotech	1/1000
IL-1β	AF5103, Affinity Biotech	1/1000
TNF-α	AF7014, Affinity Biotech	1/1000
Bcl-2	AF6139, Affinity Biotech	1/1000
Caspase-3	AF6311, Affinity Biotech	1/1000
Beta actin	sc-47778, Santa Cruz	1/2000

### Cytokine measurement

The levels of IL-1β, TNF-α, and IL-8 were measured in sera samples using commercially available ELISA kits (Rat IL-1β: YL Biont Cat No: YLA0030RA; Rat TNF-α: BT Lab Cat No: E0764Ra; Rat IL-8: BT Lab Cat No: E1167Ra) according to the manufacturer’s instructions. The absorbance was read at 450 nm using a microplate reader, and the concentrations were calculated based on standard curves.

### Oxidative stress markers

Lipid peroxidation was assessed by measuring MDA levels in lung tissue homogenates using a commercially available kit (YL Biont Cat No: YLA0029RA). Antioxidant status was evaluated by determining GSH and SOD) levels using respective kits (GSH: YL Biont Cat No: YLA0121RA; SOD: YL Biont Cat No: YLA0115RA) following the manufacturer’s protocols. Tissue protein concentrations were determined using Bradford Reagent (EchoTech Biotechnology, Cat No: BR05).

### Statistical analysis

Statistical comparisons among groups were performed using one-way analysis of variance (ANOVA) followed by Tukey’s post hoc test. A *p*-value of less than 0.05 was considered statistically significant. Histopathology was conducted using the GraphPad Prism software program (version 8.0), and data were expressed as mean ± standard deviation (SD). Cytokines and oxidative stress parameters were compared using the SPSS software program (version 25.0, IBM, USA), and data were expressed as mean ± standard standard deviation (SD).

## Results

### Histopathological findings

Histopathological examination of lung tissues revealed no significant differences in the histopathological scores among the control, D-Car 10, and D-Car 20 groups (*p* > 0.05). The LPS group exhibited significantly higher histopathological scores than the control group (*p* < 0.001). The LPS + D-Car 10 and LPS + D-Car 20 groups showed significantly lower histopathological scores than the LPS group (*p* < 0.05). Representative microscopic images of lung tissues from all groups are presented in Fig. 1, and the histopathological score values are shown in Fig. 2.

### D-carvone modulates protein expression in LPS-induced lung injury

**Fig. 3:**
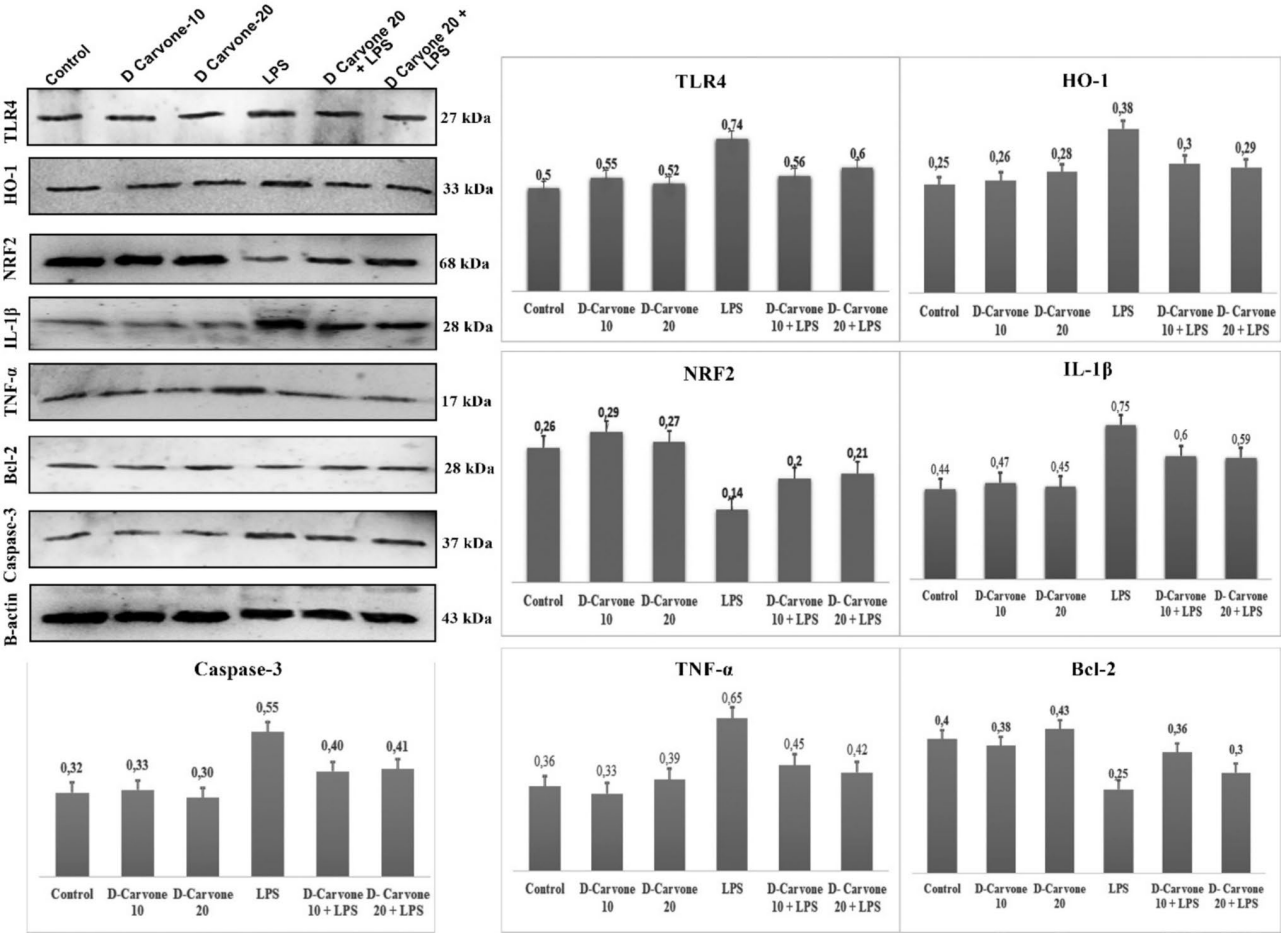
Relative expression of proteins for TLR4, HO-1, NRF2, IL-1β, TNF-α, Bcl-2, and Caspase-3. The values are given as mean ± SD

In the protein expression analysis, TLR4, HO-1, IL-1β, TNF-α, and Caspse-3 protein expressions were the highest in the LPS group. However, these expressions decreased in the D-carvone 10 + LPS and D-carvone 20 + LPS groups compared to the LPS group. The lowest protein expressions were determined in the control, D-carvone 10, and D-carvone 20 groups. While the lowest level was determined in the NRF2 and Bcl-2 protein expressions in the LPS-applied group, it was determined that this expression level increased with D-carvone 10 and D-carvone 20 applications. In addition, the highest expressions were determined in the groups with control, D-carvone 10, and D-carvone 20 applications. Expression profiles of all groups are presented in Fig. 3.

### D-carvone decreases the production of pro-inflammatory cytokines in LPS-induced lung injury

**Fig. 4:**
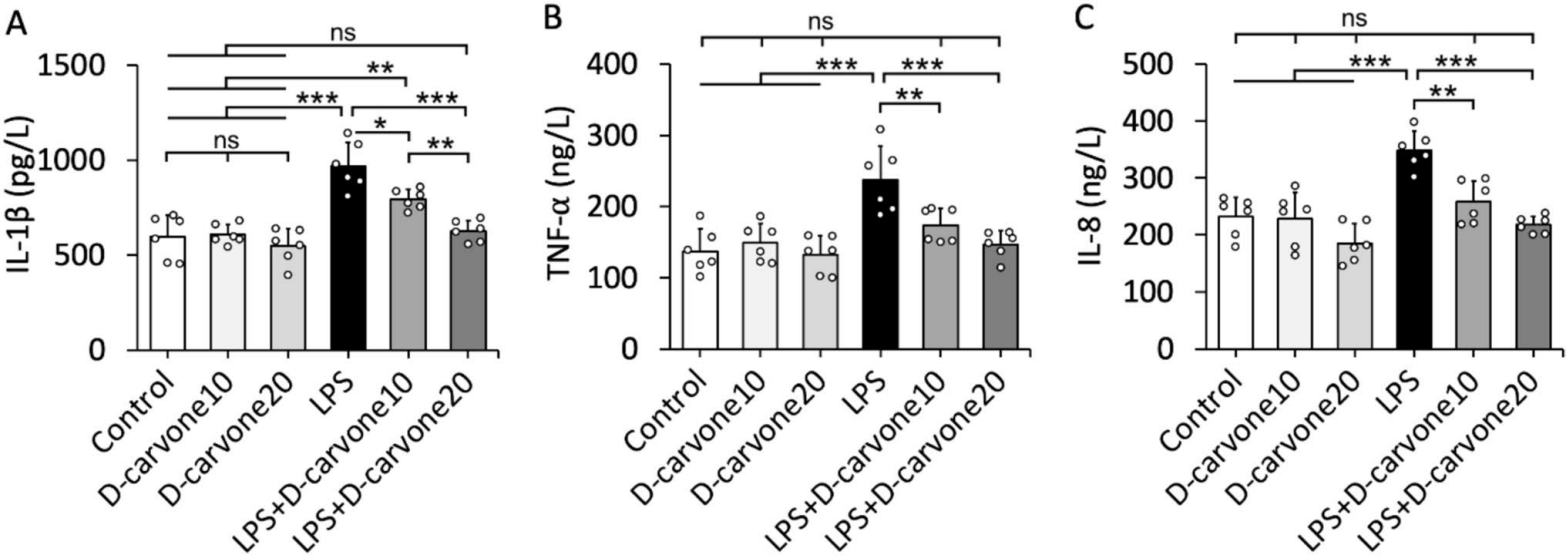
Cytokine levels. Bars are mean ± SD. Scatterplots are individual data representing the exact n number. *p < 0.05, **p < 0.01, ***p < 0.001; ns, no significant (*p* > 0.05). Statistical comparisons among groups were analyzed using one-way ANOVA with Tukey’s post hoc test

The levels of pro-inflammatory cytokines IL-1β, TNF-α, and IL-8 were significantly elevated in the lung tissues of the LPS group compared to the control group (*p* < 0.001). Treatment with D-carvone at both doses (10 and 20 mg/kg) significantly reduced the levels of these cytokines in the LPS + D-Car groups compared to the LPS group (*p* < 0.05). No significant differences were observed among the Control, D-Car 10, and D-Car 20 groups (Fig. 4).

### D-carvone alleviates oxidative stress markers in LPS-induced lung injury

**Fig. 5:**
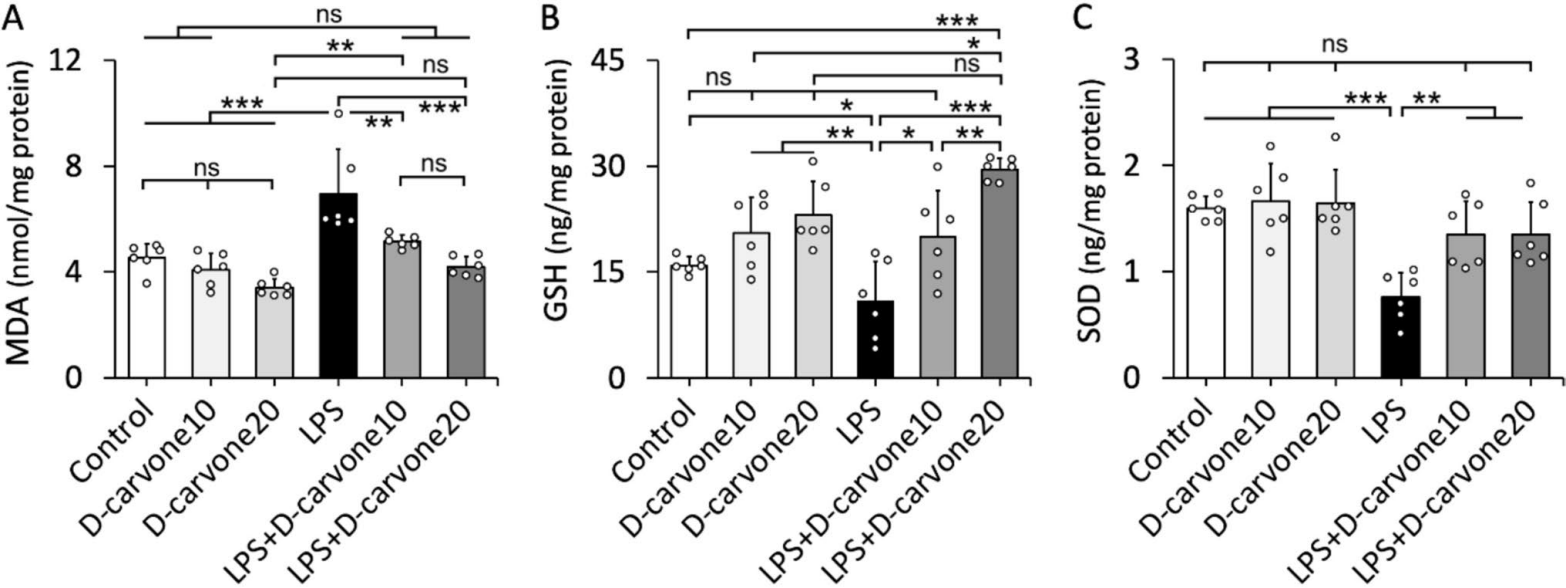
Oxidative and antioxidative status. Bars are mean ± SD. Scatterplots are individual data representing the exact n number. *p < 0.05, **p < 0.01, ***p < 0.001; ns, no significant (*p* > 0.05). Statistical comparisons among groups were analyzed by using one-way ANOVA with Tukey’s post hoc test

LPS administration significantly increased MDA levels and decreased GSH and SOD levels in lung tissues compared to the control group (*p* < 0.001), indicating oxidative stress. D-carvone treatment at both doses significantly attenuated these changes, with decreased MDA levels and increased GSH and SOD levels in the LPS + D-Car groups compared to the LPS group (*p* < 0.05). The control, D-Car 10, and D-Car 20 groups showed similar levels of these oxidative stress markers (Fig. 5).

## Discussion

In the present study, we investigated the protective effects of D-carvone against lipopolysaccharide (LPS)-induced acute lung injury (ALI) in a rat model. Our findings demonstrate that D-carvone administration significantly attenuated LPS-induced lung damage, as evidenced by the reduction in histopathological changes, inflammatory response, oxidative stress, and apoptosis.

Histopathological examination of lung tissues revealed that LPS administration caused significant lung injury, characterized by pulmonary edema, alveolar septal thickening, and inflammatory cell infiltration. These findings are consistent with previous studies that have reported similar histopathological changes in LPS-induced ALI models (Xie et al. 2012; Jiang et al. 2018). However, treatment with D-carvone at doses of 10 and 20 mg/kg significantly attenuated these pathological changes, suggesting its potential therapeutic value in ALI. The protective effects of D-carvone against LPS-induced lung injury are mediated through its anti-inflammatory and antioxidant properties.

Inflammation plays a central role in the pathogenesis of ALI. LPS, a potent inducer of inflammation, binds to TLR4 on immune cells, triggering the activation of downstream signaling pathways, including NF-κB and MAPK (Lu et al. 2008). This leads to the production of pro-inflammatory cytokines, such as IL-1β, TNF-α, and IL-8 in LPS-induced lung injury (Ozkanlar et al. 2023), which recruit neutrophils to the lungs and exacerbate tissue damage (Matthay et al. 2019). Indeed, the TNF-α and IL-1β are strongly related with an acute response to a clinical disease along with the recruitment of the leucocytes (Ulas et al. 2023). In our study, D-carvone downregulated the expression of TLR4, IL-1β, and TNF-α in LPS-induced lung tissue. These findings suggest that D-carvone may inhibit the TLR4-mediated inflammatory signaling pathway, thus reducing inflammation in the lungs. This is in line with previous studies that have reported the anti-inflammatory effects of D-carvone in various experimental models (Dai et al. 2020; Zhao and Du 2020). To further confirm the anti-inflammatory effects of D-carvone, we measured the levels of pro-inflammatory cytokines IL-1β, TNF-α, and IL-8 in lung tissues. Our results showed that D-carvone treatment significantly reduced the levels of these cytokines in LPS-induced lung tissue, which is in line with the Western blot findings. These cytokines play a crucial role in the pathogenesis of ALI by recruiting inflammatory cells, increasing vascular permeability, and promoting alveolar damage (Bhatia et al. 2009). Therefore, the ability of D-carvone to suppress these cytokines may contribute to its protective effects against LPS-induced lung injury.

Oxidative stress is another key factor contributing to the pathogenesis of ALI. LPS-induced inflammation leads to the excessive production of ROS, which cause lipid peroxidation, protein oxidation, and DNA damage, ultimately leading to cell death and organ dysfunction (Kellner et al. 2017). Recent studies have demonstrated that various therapeutic agents can protect against oxidative stress-induced tissue damage through different mechanisms (Rajeshwari & Raja 2015a; Dai et al. 2020; Celep et al. 2024). Oxidative stress parameters, along with inflammatory findings, are used to evaluate the response of septic lung tissues (Üstündağ et al. 2023a, 2023b). D-carvone has shown significant antioxidant properties across various disease models through multiple mechanisms. Zhu et al. demonstrated that D-carvone (40 mg/kg) enhanced antioxidant enzyme status while reducing oxidative stress and pro-inflammatory markers, suggesting a coordinated regulation of antioxidant defense systems (Zhu et al. 2020). This is further supported by Rajeshwari et al., who reported that D-carvone restored key antioxidant enzymes including SOD, CAT, and GPx activities in hypertensive rats, indicating its ability to modulate multiple antioxidant pathways (Rajeshwari and Raja 2015b). Additionally, Petchi et al. demonstrated that D-carvone treatment significantly increased ROS production and decreased antioxidant levels in leukemic cells, suggesting its selective oxidative stress modulation in different tissue types (Iyappan et al. 2021). Consistent with these findings, our study showed that D-carvone treatment significantly attenuated LPS-induced oxidative stress in lung tissues, as demonstrated by the decreased MDA levels and increased GSH and SOD levels. The restoration of these antioxidant parameters suggests that D-carvone’s protective effects may be mediated through the enhancement of cellular antioxidant defense mechanisms, thereby breaking the cycle of oxidative damage and inflammation in ALI pathogenesis. This is consistent with previous reports on the antioxidant effects of D-carvone in other diseases (Zhao and Du 2020; Ogaly et al. 2022).

Apoptosis, a form of programmed cell death, has been implicated in the pathogenesis of ALI (Galani et al. 2010). LPS-induced inflammation and oxidative stress can trigger apoptosis in lung epithelial and endothelial cells, leading to the disruption of the alveolar-capillary barrier and impaired gas exchange (Albertine et al. 2002). The balance between pro-apoptotic and anti-apoptotic proteins plays a crucial role in determining cell survival during inflammatory conditions. In our study, Western blot analyses showed that D-carvone upregulated the expression of the anti-apoptotic protein Bcl-2 while downregulating the expression of the pro-apoptotic protein caspase-3 in LPS-induced lung tissue. This protective effect aligns with recent findings by Mohamed et al., who demonstrated that D-carvone pretreatment significantly reduced multiple apoptotic markers including caspase-1, −3, and −9, while elevating Bcl-2 expression in hepatic ischemia–reperfusion injury (Mohamed and Younis 2022). Notably, Lv et al. demonstrated that D-carvone can induce apoptosis through JAK/STAT3 pathway inhibition and ROS production in gastric cancer cells, while exhibiting protective anti-apoptotic effects in normal tissue (Lv et al. 2021). This differential regulation suggests that D-carvone’s effects on apoptotic pathways are tissue-specific and may depend on the underlying pathophysiological state. In our ALI model, D-carvone’s ability to regulate the Bcl-2/Caspase-3 axis appears to protect against excessive inflammatory-induced apoptosis, thereby helping maintain alveolar-capillary barrier integrity. These findings indicate that D-carvone’s anti-apoptotic effects in inflammatory conditions represent a key mechanism in its tissue-protective properties, particularly in maintaining normal cellular homeostasis (Dai et al. 2020; Erbaş et al. 2024b).

The Nrf2 is a key transcription factor that regulates the expression of antioxidant and cytoprotective genes (Cho and Kleeberger 2014). Under normal conditions, Nrf2 is sequestered in the cytoplasm by Kelch-like ECH-associated protein 1 (Keap1). However, under oxidative stress, Nrf2 dissociates from Keap1 and translocates to the nucleus, where it binds to antioxidant response elements (AREs) and promotes the transcription of antioxidant enzymes, such as heme oxygenase-1 (HO-1) (Loboda et al. 2016). In our study, analyses showed that D-carvone upregulated the expression of Nrf2 and HO-1 in LPS-induced lung tissue. These findings suggest that D-carvone may activate the Nrf2-dependent antioxidant response, thus reducing oxidative stress and inflammation in the lungs. A recent mechanistic study has demonstrated that (R)-(-)-carvone exhibits its anti-inflammatory effects through dual mechanisms: direct inhibition of JNK1 phosphorylation and enhancement of nuclear Nrf2 accumulation, which collectively suppress NF-κB transcriptional activity (Sousa et al. 2023). Moreover, Ogaly et al. revealed that D-carvone’s protective effects are also mediated through modulation of the TGF-β1/SMAD3 pathway, which synergizes with Nrf2 activation to attenuate tissue injury (Ogaly et al. 2022). This interplay between Nrf2 activation, NF-κB suppression, and TGF-β1 signaling represents key mechanisms by which D-carvone modulates inflammatory responses and oxidative stress in various tissue injury models. The findings of this study are consistent with previous reports on the anti-inflammatory and antioxidant effects of D and/or L-carvone in other disease models (Chen et al. 2020; Dai et al. 2020; Asle-Rousta et al. 2023; Mahde and Kathem 2023). Similar protective effects against tissue damage have been observed in various experimental models using different therapeutic agents (Celep et al. 2023, Kara and Ozkanlar 2023; Ozkanlar et al. 2023). The protective effects of D-carvone appear to be mediated through multiple mechanisms, including the inhibition of TLR4-mediated inflammatory signaling, activation of Nrf2-dependent antioxidant response, suppression of apoptosis, and reduction of pro-inflammatory cytokine levels.

## Conclusion

In conclusion, this study demonstrates that D-carvone exerts protective effects against LPS-induced acute lung injury in rats through multiple molecular mechanisms. Our findings reveal that D-carvone’s therapeutic effects are mediated through three key pathways: (1) suppression of the TLR4/NF-κB inflammatory signaling cascade, resulting in reduced pro-inflammatory cytokine production (IL-1β, TNF-α, IL-8); (2) activation of the Nrf2/HO-1 antioxidant pathway, leading to enhanced antioxidant defense mechanisms and reduced oxidative stress markers; and (3) modulation of the Bcl-2/Caspase-3 axis, promoting cell survival and preventing excessive apoptosis in lung tissue. These molecular mechanisms were further supported by histopathological findings, which showed that D-carvone treatment significantly reduced inflammatory cell infiltration, alveolar septal thickening, and maintained normal lung architecture. Collectively, these findings suggest that D-carvone could be a promising therapeutic candidate for ALI prevention and treatment. However, further studies are warranted to validate these results and explore their clinical potential, particularly focusing on optimal dosing strategies and potential combination therapies for enhanced therapeutic efficacy.

## Data Availability

All source data for this work (or generated in this study) are available upon reasonable request.
